# Night Sleep Duration and Risk of Incident Anemia in a Chinese Population: A Prospective Cohort Study

**DOI:** 10.1038/s41598-018-22407-5

**Published:** 2018-03-05

**Authors:** Xiaoxue Liu, Qiaofeng Song, Wanning Hu, Xiaochen Han, Jianhui Gan, Xiang Zheng, Xizhu Wang, Shouling Wu

**Affiliations:** 1 Department of Cardiology, Tangshan People’s Hospital, North China University of science and technology, Tangshan, China; 2 The Cancer Institute, Tangshan People’s Hospital, Tangshan, China; 3 Department of Head and Neck Surgery, Tangshan People’s Hospital, Tangshan, China; 4 Department of Anesthesia, Tangshan People’s Hospital, Tangshan, China; 5 Department of Nuclear magnetic resonance, Tangshan People’s Hospital, Tangshan, China; 6 Department of Cardiology, Kailuan Hospital, North China University of science and technology, Tangshan, China

## Abstract

The purpose was to study the association between sleep duration and the prevalence of anemia in Chinese people. There were 84,791 participants (men: 79.1%; women: 20.9%) aged 18–98 years in the prospective study. We divided the participants into five categories based on the individual sleep duration: ≤5 h, 6 h, 7 h(reference), 8 h, and ≥9 h. Anemia was defined based on hemoglobin <12 g/dL for men and <11 g/dL for women. The Cox proportional hazards model was used to assess the association between sleep duration and anemia. During median follow-up of 7.9 years, 2698 cases of anemia had occurred. The HRand (95% CI) of anemia (7 h as the reference group) for individuals reporting ≤5 h, 6 h, 8 h, and ≥9 h were 1.23(1.04–1.45), 1.26(1.11–1.44), 1.04(0.92–1.16) and 1.42(1.08–1.86), respectively. It showed that there was a significant interaction on the risk of anemia between sleep duration and sex in the secondary analysis (p < 0.001).The significant association between long sleepduration and anemia was found in women (HR, 2.29; 95% CI, 1.56–3.37), not in men(HR, 0.90; 95% CI, 0.60–1.34). Both short and long night sleep duration were associated with increased risk of anemia.

## Introduction

Anemia has been linked to cardiovascular disease and mortality^1–6^. Similar with the common cardiovascular risk factors, such as smoking, diabetes, hypertension and hypercholesterolemia, anemia can also increase the risk of mortality, morbidity and hospitalization^1–6^. Consequently, anemia has lately been characterized as another cardiovascular risk factor^7–9^. Thus, it is necessary to identify anemia-related risk factors so that. we can take effective prevention and management strategies as early as possible. In addition to the traditional risk factors including age10, malnutrition, chronic kidney disease11, and poor glycemic control^12,13^, we have found some new risk factors for anemia, such as sleep^14,15^.

Recently, many epidemiological studies have reported that either a long or a short sleep duration is independently associated with cardiovascular disease^16–22^.Both sleep duration and anemia are recognized as strong, independent risk factors for ischemia and mortality events^23,24^.However, only a few studies to date have examined the association between sleep alterations and risk of iron deficiency anemia in infancy^14,15^. Another study evaluated the association between self-reported sleep duration and anemia on British people over 50 years old. The result showed that short sleep time could lead to low hemoglobin concentration, and disturbed sleep also increased the risk of anemia^25^. It is limited on the association between night sleep duration and risk for anemia in the general population. Alternatively, considering that a women-specific associations of short sleep duration with hypertension were reported in British population^26,27^, we further conducted a longitudinal analysis focusing primarily on the association between sleep duration and anemia, using the comprehensive data from the Kailuan Study^28^ stratified by age and sex.

## Results

The percent of participants who reported sleeping for ≤5 h, 6 h, 7 h, 8 h, and ≥9 h per night were 7.1%, 17.3%, 17.8%, 60.0%, and 1.8%, respectively. The baseline characteristics by sleep duration was shown in Table 1. There were significant associations between sleep duration and age, sex, education level, smoking status, drinking status, physical activity, body mass index, blood pressure level, fasting blood glucose, total cholesterol, hypertension, diabetes mellitus, dyslipidemia, snoring status, and high sensitive C-reactive protein. The similar result was also found in our previous paper^29^.

**Table 1:** Baseline characteristics according to sleep duration.

Variable	Sleep duration(hours)					p value
	≤5 (n = 5998)	6.0 (n = 14697)	7 (n = 15103)	8.0 (n = 47481)	≥9 (n = 1512)	
**Questionnaire-based data**						
Age, years	54.67 ± 11.36	52.24 ± 11.60	50.34 ± 12.17	49.58 ± 11.88	49.02 ± 13.87	<0.01
Sex male, n (%)	4946 (82.46)	12377 (84.21)	12169 (80.57)	36398 (76.66)	1153 (76.26)	<0.01
High school or above, n (%)	1105 (18.42)	3527 (24.00)	4765 (31.55)	7939 (16.72)	468 (30.95)	<0.01
Current smoker, n (%)	3402 (56.72)	8636 (58.76)	8445 (55.92)	13173 (27.74)	842 (55.69)	<0.01
Current alcohol, n (%)	3474 (57.92)	8974 (61.06)	8870 (58.73)	13458 (28.34)	835 (55.22)	<0.01
Active physical-activity level, n (%)	1535 (25.59)	3456 (23.52)	3523 (23.33)	4475 (9.42)	290 (19.18)	<0.01
Snoring, n (%)	1576 (26.28)	3277 (22.30)	2936 (19.44)	4089 (8.61)	293 (19.38)	<0.01
**Exam-based data**						
Hypertension, n (%)	2951 (49.20)	6438 (43.80)	5992 (39.67)	20347 (42.85)	602 (39.81)	<0.01
Systolic blood pressure, mmHg	132.89 ± 21.25	130.90 ± 20.39	129.23 ± 20.47	130.04 ± 20.64	128.44 ± 21.87	<0.01
Diastolic blood pressure, mmHg	84.09 ± 11.76	83.38 ± 11.38	82.60 ± 11.37	83.61 ± 11.83	82.08 ± 12.22	<0.01
Diabetes mellitus, n (%)	698 (11.64)	1418 (9.65)	1333 (8.83)	3920 (8.26)	154 (10.19)	<0.01
Fasting blood glucose, mmol/L	5.51 ± 1.74	5.46 ± 1.62	5.43 ± 1.51	5.45 ± 1.63	5.48 ± 1.74	0.22
Dyslipidemia, n (%)	2481 (41.36)	5699 (38.78)	5610 (37.14)	15749 (33.17)	566 (37.43)	<0.01
Total cholesterol, mmol/L	5.02 ± 1.17	4.99 ± 1.21	4.96 ± 1.21	4.94 ± 1.11	4.89 ± 1.16	<0.01
Triglycerides, mmol/L	1.68 ± 1.31	1.71 ± 1.42	1.68 ± 1.36	1.67 ± 1.37	1.68 ± 1.37	0.35
Low-density lipoprotein, mmol/L	2.46 ± 0.87	2.46 ± 0.87	2.46 ± 0.87	2.31 ± 0.85	2.36 ± 0.87	<0.01
High-density lipoprotein, mmol/L	1.55 ± 0.42	1.55 ± 0.40	1.52 ± 0.39	1.55 ± 0.40	1.53 ± 0.39	<0.01
Body, mass index, kg/m^2^	25.04 ± 3.44	25.13 ± 3.41	25.09 ± 3.42	25.05 ± 3.50	25.09 ± 3.60	<0.05
High sensitivity C-reactive protein, mg/L	0.80 (0.31–2.00)	0.78 (9.30–1.89)	0.80 (0.30–1.90)	0.73 (0.28–2.00)	0.88 (0.30–2.10)	<0.01

Age, the percentage of women, education level, and the level of sensitivity C-reactive protein among participants with anemia were higher than those without anemia. In contrast, the prevalence of hypertension, the prevalence of obesity and snoring prevalence were lower among participants with anemia than without anemia (all *p* < 0.001).(Table 2)

**Table 2:** Comparisons between patients with and without anemia among Kailuan study.

Variable	Anemia	Without anemia	p value
No.	2698	82093	
**Questionnaire-based data**			
Age, years	50.55 ± 13.36	50.52 ± 11.93	0.008
Sex male, n (%)	1770 (65.60)	65273 (79.51)	<0.0001
High school or above, n (%)	627 (23.24)	17177 (20.92)	<0.05
Current smoker, n (%)	882 (32.69)	33616 (40.95)	<0.0001
Current alcohol, n (%)	970 (35.95)	34641 (42.20)	<0.0001
Active physical-activity level, n (%)	347 (12.86)	12932 (15.75)	<0.0001
Snoring, n (%)	328 (12.16)	11843 (14.43)	<0.0001
**Exam-based data**			
Hypertension, n (%)	1024 (37.95)	35306 (43.01)	<0.0001
Systolic blood pressure, mmHg	127.64 ± 21.64	130.30 ± 20.62	<0.0001
Diastolic blood pressure, mmHg	81.40 ± 11.73	83.46 ± 11.68	<0.0001
Diabetes mellitus, n (%)	244 (9.04)	7279 (8.87)	0.750
Fasting blood glucose, mmol/L	5.49 ± 1.93	5.45 ± 1.60	0.291
Dyslipidemia, n (%)	912 (33.80)	29193 (35.56)	0.060
Total cholesterol, mmol/L	4.93 ± 1.12	4.96 ± 1.15	<0.05
Triglycerides, mmol/L	1.53 ± 1.19	1.68 ± 1.38	<0.0001
Low-density lipoprotein, mmol/L	2.23 ± 1.07	2.38 ± 0.85	<0.0001
High-density lipoprotein, mmol/L	1.54 ± 0.40	1.54 ± 0.40	0.142
Body, mass index, kg/m^2^	24.47 ± 3.56	25.09 ± 3.46	<0.0001
High sensitivity C-reactive protein, mg/L	0.94 (0.29–3.10)	0.76 (0.30–1.90)	<0.0001

As shown in Table 3, we can observe the hazard ratios for anemia according to sleep duration in total population and stratified by sex. Out of all the 84791 individuals, 2,698 participants developed anemia (men: 1,770; women: 928). The incidence per 1000 person years of anemia was 3.8 in men, and 7.2 in women. In the COX regression analysis, with adjustment for all variables (model 3), the multivariable adjusted hazard ratios of anemia among the participants were 1.23 (95% CI, 1.04–1.45) for a ≤ 5 h sleep duration, 1.26 (95% CI, 1.11–1.44) for 6 h, 1.04(95% CI, 0.92–1.16) for 8 h and 1.42 (95% CI, 1.08–1.86) for ≥9 h compared with the participants with 7 h of sleep. The risk of anemia in women with more than 8 hours of sleep (HR, 2.29; 95% CI, 1.56–3.37) was higher, but not in men (HR, 0.90; 95% CI, 0.60–1.34), a formal test for difference by sex also found statistical significance (p-interaction for long sleep duration <0.001; P-interaction for short sleep duration >0.05). In addition, the association between sleep duration and anemia was still significant in participants excluding the individuals who have myocardial infarction, stroke and cancer.

**Table 3:** Hazard ratios (95% CI) for anemia according to sleep duration in the Kailuan Study.

**Total**	**Sleep duration(hours)**				
	≤5	6	7	8	≥9
Case(incidence per 1000 person years)	204 (4.93)	510 (4.94)	431 (4.01)	1490 (4.47)	63 (6.08)
Model 1	1.22 (1.03–1.44)	1.26 (1.10–1.43)	reference	1.08 (0.97–1.20)	1.45 (1.11–1.89)
Model 2	1.24 (1.04–1.46)	1.26 (1.11–1.44)	reference	1.03 (0.92–1.15)	1.45 (1.11–1.88)
Model 3	1.23 (1.04–1.45)	1.26 (1.11–1.44)	reference	1.04 (0.092–1.16)	1.42 (1.08–1.86)
Sensitivity analysis*	1.20 (1.01–1.43)	1.22 (1.07–1.39)	reference	1.01 (0.90–1.13)	1.36 (1.03–1.80)
Sensitivity analysis^†^	1.18 (1.02–1.38)	1.22 (1.10–1.36)	0.97 (0.86–1.08)	reference	1.37 (1.06–1.78)
**Women** ^§^					
Case(incidence per 1000 person years)	41 (5.34)	114 (6.73)	116(5.40)	622 (7.83)	35 (14.03)
Model 1	1.20 (0.84–1.72)	1.37 (1.06–1.78)	reference	1.34 (1.10–1.63)	2.22 (1.52–3.25)
Model 2	1.24 (0.86–1.77)	1.39 (1.08–1.80)	reference	1.26 (1.03–1.55)	2.20 (1.50–3.21)
Model 3	1.20 (0.84–1.73)	1.33 (1.02–1.74)	reference	1.25 (1.02–1.54)	2.29 (1.56–3.37)
Sensitivity analysis*	1.14 (0.78–1.68)	1.34 (1.02–1.76)	reference	1.26 (1.02–1.55)	2.37 (1.61–3.48)
**Men** ^§^					
Case(incidence per 1000 person years)	163 (4.84)	396 (4.59)	315(3.66)	868 (3.42)	28 (3.56)
Model 1	1.23 (1.02–1.49)	1.22 (1.05–1.41)	reference	0.93 (0.82–1.06)	0.95 (0.65–1.40)
Model 2	1.24 (1.02–1.50)	1.22 (1.05–1.42)	reference	0.89 (0.77–1.02)	0.95 (0.65–1.40)
Model 3	1.23 (1.02–1.49)	1.23 (1.06–1.43)	reference	0.90 (0.78–1.03)	0.90 (0.60–1.34)
Sensitivity analysis*	1.21 (1.00–1.47)	1.18 (1.01–1.37)	reference	0.86 (0.75–1.00)	0.79 (0.51–1.21)
P for interaction	0.168	0.777		<0.001	<0.001

Model 1: Adjusted for age and sex.

Model 2: Adjusted for age, sex, level of education, smoking, alcohol, physical activity, and snoring.

Model 3: Adjusted for variables in Model 2 plus hypertension, diabetes mellitus, dyslipidemia, body mass index,

and high-sensitivity C-reactive protein.

*Adjusted for Model 3 and further excluded individuals with myocardial infarction, stroke and cancer.

^§^Adjusted for above confounding factors without sex.

^†^Adjusted for Model 3 and used 8 hours/night as the reference group.

Further study stratified by different age groups was analysed in Table 4. Participants aged <60 years and who slept ≤5 hours (HR, 1.24; 95% CI, 1.01–1.53) or ≥9 hours (HR, 1.40; 95% CI, 1.04–1.90) were found likely to develop anemia. The older participants (ages ≥ 60) who slept ≤5 h (HR, 1.16; 95% CI, 0.86–1.56) or ≥9 hours(HR, 1.04; 95% CI, 0.57–1.89) were less likely to develop anemia. The interaction of sleep duration with age on the incident anemia is not significant (p > 0.05).

**Table 4:** Hazard ratios (95% CI) for anemia according to sleep duration stratified by age in the Kailuan Study.

	**Sleep duration(hours)**				
	≤5	6.0	7	8.0	≥9
**Age** < **60**					
Case(incidence per 1000 person years)	128 (4.28)	376 (4.67)	326 (3.73)	1177 (4.18)	51 (6.08)
Model 1	1.27 (1.03–1.56)	1.36 (1.17–1.58)	reference	1.05 (0.93–1.19)	1.43 (1.07–1.93)
Model 2	1.26 (1.02–1.54)	1.35 (1.16–1.56)	reference	1.00 (.088–1.14)	1.43 (1.06–1.92)
Model 3	1.24 (1.01–1.53)	1.35 (1.16–1.57)	reference	1.02 (0.89–1.16)	1.40 (1.04–1.90)
**Age** ≥ **60**					
Case(incidence per 1000 person years)	76 (6.60)	134 (5.89)	105 (5.24)	313 (6.06)	12 (6.12)
Model 1	1.17 (0.87–1.57)	1.10 (0.85–1.42)	reference	1.11 (0.89–1.39)	1.04 (0.57–1.90)
Model 2	1.17 (0.87–1.58)	1.10 (0.86–1.43)	reference	1.02 (1.81–1.29)	1.04 (0.57–1.89)
Model 3	1.16 (0.86–1.56)	1.07 (0.83–1.39)	reference	1.01 (0.80–1.27)	1.04 (0.57–1.89)
P for interaction	0.704	0.633		0.302	0.579

Model 1: Adjusted for age and sex.

Model 2: Adjusted for age, sex, level of education, smoking, alcohol, physical activity, and snoring.

Model 3: Adjusted for variables in Model 2 plus hypertension, diabetes mellitus, dyslipidemia, body mass index,

and high-sensitivity C-reactive protein.

## Discussion

In the present study, both long and short sleep durations independently predicted an increased risk for incident anemia, after a follow-up of median 7.9 years, as shown during a median 7.9 years of follow-up. These relationships persist even after adjusting other known major risk factors, such as smoking, drinking, diabetes, hypertension, dyslipidemia, obesity, and high-sensitivity C-reactive protein. Sensitivity analyses further confirmed these findings.

The English Longitudinal Study of Ageing (ELSA)^25^, with participants of 6465 men and women aged 50–99 years, found that there was significant influence of short and disturbed sleep on low hemoglobin concentrations. Results of this study further found that the disturbed sleep was a risk factor of anemia.Our results are partly consistent with this study^25^.But the difference is that we also demonstrated that a long sleep duration was an independent predictor for incident anemia. Additionally, in the previous studies, traditional predictors for anemia had no significant difference between men and women.^25^. However, we found that the risk of anemia in women with long sleep duration was higher. But the difference was not significant among men. We have not found the exact cause of the result yet. The reason for the gender difference in the relationship between the sleep time and the anemia may be due to the differences in hormonal secretion and psychological factors in gender. Unfortunately, we did not collect sufficient data on the pre- or post-menopause status of women participants, which appeared to be an important determinant of anemia risk in women. In addition, considering that this connection might be affected by different sleep behaviors in different age groups, we performed a stratified analysis based on age. Participants aged <60 years and sleepping ≥9 hours were found to be more likely to develop anemia.However, there was no interaction between sleep duration and age in the risk of anemia (p-interaction >0.05).The above results stratified by age and sex further endorsed the possibility that the associations observed may be driven entirely by younger women.However, the lack of information on biological differences among different groupslimits us to further investigate whether the association could be modified or mediated by these factors.

Previous studies found that sleep apnea might be another pathway mediating long sleep duration with chronic diseases^30^. Evidence also have showed that sleep apnea may be an important anemia predictor^31–34^. A cohort study conducted in children also showed that children with sickle cell anemia had a high prevalence of sleep apnea with typical symptoms^31^. Unfortunately, sleep apnea was not measured in our study, but snoring status was used as a confounder instead of sleep apnea. After adjusting snoring status, sleep duration in our study was persistently associated with incident anemia.

We have not found the underlying mechanism for sleep duration and incident anemia. Inflammation is one of the most important biological pathways, because the long sleep time can lead to the increasing of inflammatory markers. In addition, the result that sleep deprivation could cause an increase in inflammatory response has been shown by a recent study^35^. And in this study, individuals who reported short(≤5 h) or long sleep duration (≥9 h)were more likely to be engaged in higher level of sensitivity C-reactive protein group than those who slept 7 h. We also found that the level of sensitivity C-reactive protein in participants with anemia was higher than those without anemia.

### Limitations

First, we collect the data of sleep duration through self-reported questionnaires. In contrast, the polysomnography is a more valid and reliable measurement of sleep. Information on Chinese midday naps and sleep quality were not collected in current study. Participants with sleep apnea were not excluded, which is associated with high risk of anemia^31,32^. However, we adjusted for snoring status as an alternative confounder. In addition, the full model in our study was adjusted for corresponding risk factors for sleep apnea, such as body mass index, age, and smoking^36^. Second, anemia in our study was only diagnosed using the hemoglobin content without employing red cell hematocrit, mean cellular volume, and bone marrow iron staining. Therefore, we could not distinguish different types of anemia (including sickle cell anemia, iron deficiency anemia or renal anemia) in this study. Third, we only investigated the sleep duration at the baseline, without taking the sleep duration changes into consideration. Indeed, any subsequent alterations in sleep may lead to a non-differential misclassification and potentially underestimate the sleep–anemia association^14^. Fourth, there was not a rationale forthe analysis in our studyusing 60 years as a cut-off value. Alternatively, we used the same cut-off valueof 60 in our previous publications^20,37^. Finally, we only investigated employees of the Kailuan Coal Company, which most of them were men. Therefore, the results may not be applicable to the general population.

In conclusion, our study suggest that both long and short sleep durations may cause an increased risk of anemia in a Chinese population. In addition to nutritional deficiencies, malignant tumor or other chronic illnesses, inappropriate sleep might be taken in this condition.

## Methods

### Ethics statement

The protocol for the present study was approved by the Ethics Committee of Kailuan General Hospital in compliance with the Declaration of Helsinki, and all participants provided written informed consent.^29,38^.

### Study design and participants

The Kailuan study was a prospective cohort study involving 101510 participants (men: 81110; women: 20400, aged 18–98 years) in Kailuan community from June 2006 to October 2007^29^. This study enrolled 84791 participants, excluding someone who had history of anemia (3703), incomplete sleep data (3986), and incomplete hemoglobin data (9030). We carried out questionnaire surveys and investigated clinical and laboratory indicators among all the participants. Before the study, all doctors and nurses received rigorous unified training.

### Assessment of sleep duration

Sleep duration data was collected through a self-reported answer to the question “How many hours of sleep have you had on an average night in the preceding 3 months?”We divided sleep durations into five groups according to the responses: ≤5 hours, 6 hours, 7 hours, 8 hours, and ≥9 hours. Additionally, participants were asked to answer “yes” or “no” to the question “Do you generally snore when you sleep?”^29^.

### Assessment of potential covariates

We collected the data of demographic and clinical characteristics self-reported questionnaires, including age, sex, alcohol use, education, and disease history. Educational status was divided into “illiterate or primary school”, “middle school”, or “high school or above”. Physical activity was divided into “ ≥80 minutes every week(active)”, “1 to 79 minutes every week (intermediate)”, or “none”.^29,38^. Smoking status and drinking status were divided into “never”, “former”, or “current”. Body mass index (BMI) was calculated as the weight (kg) divided by the square of height (meters2). Blood pressure was measured three times using a standardized sphygmometer in the seated position. We used the average at last.

We measured the levels of triglyceride (TG), total cholesterol (TC), low-density lipoprotein cholesterol (LDL-C), high-density lipoprotein cholesterol (HDL-C), fasting blood-glucose (FBG), and high-sensitivity C-reactive protein (HCRP),. All these blood samples were analyzed by using a Hitachi 747 auto-analyzer (Hitachi; Tokyo, Japan)^29^ Diabetes was defined as having a history of diabetes, the use of glucose-lowering agents, or a fasting blood glucose ≥7 mmol/l. Hypertension was defined as systolic blood pressure ≥140 mmHg, diastolic blood pressure ≥90 mmHg, having a history of hypertension and/or the use of antihypertensive agents. Dyslipidemia was defined as having a history of dyslipidemia or the use of anti-lipidemic agents, TC ≥6.2 mmol/l, TG ≥2.3 mmol/l, LDL-C ≥ 4.1 mmol/l, or HDL-C < 1.0 mmol/l^39^.

### Follow-up and anemia assessment

Participants were followed up by face-to-face interviews at every 2-year routine medical examination until December 31, 2015, or until the event of interest or death^29^.Person-years were calculated from the date the 2006 interview was conducted to the date when anemia was detected, date of death, or date of the last attended interview in this analysis, whichever came first^29^. Anemia status (no/yes) was defined based on hemoglobin <12 g/ dL for men and <11 g/dL for women^40^.

### Statistical analyses

The statistical analysis was performed using SAS 9.4. We described continuous variables by their means ± standard deviations, and compared groups using one-way analysis of variance (ANOVA). Categorical variables were described as percentages and compared by the Chi-square test. We used Cox proportional hazards regression to estimate the risk of anemia by HR with 95% confidence intervals (CIs). Model 1 adjusted for age and sex. Model 2 further adjusted for level of education, smoking, alcohol, physical activity, and snoring. Model 3 further adjusted for hypertension, diabetes mellitus, dyslipidemia, body mass index, and high-sensitivity C-reactive protein.We assessed the association between sleep duration and age/sex in the secondary analyses. In addition, the robustness of our findings also be tested by a sensitivity analysis. Because major chronic illnesses including history of myocardial infarction, stroke and cancer can affect sleep behavior and future anemia risk, we repeated our analysis after excluding individuals with these conditions. Because 11 hospitals participated in the study, we used a Cox proportional hazards model with a sandwich covariance matrix as a random effect to account for the potential confounding effect of multiple hospitals participating in the study^29^. All statistical tests were two-sided, and the significance level was set at 0.05.
